# Energy-Saving Multi-Agent Deep Reinforcement Learning Algorithm for Drone Routing Problem

**DOI:** 10.3390/s24206698

**Published:** 2024-10-18

**Authors:** Xiulan Shu, Anping Lin, Xupeng Wen

**Affiliations:** 1 School of Intelligent Manufacturing Engineering, Zhanjiang University of Science and Technology, Zhanjiang 524000, China; xiulanshu8383@163.com; 2 School of Physics and Electronic Electrical Engineering, Xiangnan University, Chenzhou 423000, China; 3 China Nanhu Academy of Electronic and Information Technology, Jiaxing 314107, China; xupengwen0411@163.com

**Keywords:** energy savings, deep reinforcement learning, drone routing, multiple agents

## Abstract

With the rapid advancement of drone technology, the efficient distribution of drones has garnered significant attention. Central to this discourse is the energy consumption of drones, a critical metric for assessing energy-efficient distribution strategies. Accordingly, this study delves into the energy consumption factors affecting drone distribution. A primary challenge in drone distribution lies in devising optimal, energy-efficient routes for drones. However, traditional routing algorithms, predominantly heuristic-based, exhibit certain limitations. These algorithms often rely on heuristic rules and expert knowledge, which can constrain their ability to escape local optima. Motivated by these shortcomings, we propose a novel multi-agent deep reinforcement learning algorithm that integrates a drone energy consumption model, namely EMADRL. The EMADRL algorithm first formulates the drone routing problem within a multi-agent reinforcement learning framework. It subsequently designs a strategy network model comprising multiple agent networks, tailored to address the node adjacency and masking complexities typical of multi-depot vehicle routing problem. Training utilizes strategy gradient algorithms and attention mechanisms. Furthermore, local and sampling search strategies are introduced to enhance solution quality. Extensive experimentation demonstrates that EMADRL consistently achieves high-quality solutions swiftly. A comparative analysis against contemporary algorithms reveals EMADRL’s superior energy efficiency, with average energy savings of 5.96% and maximum savings reaching 12.45%. Thus, this approach offers a promising new avenue for optimizing energy consumption in last-mile distribution scenarios.

## 1. Introduction

Recently, significant challenges have arisen in the implementation of ‘last mile’ e-commerce logistics distribution [1]. Ensuring efficient development of this phase has emerged as a focal research area within logistics distribution. The predominant method for terminal distribution involves human-operated vehicles, contributing to traffic congestion, jeopardizing personnel safety, and consuming substantial energy. In contrast, the agility, speed, and energy efficiency of drones have prompted a heightened interest in drone distribution as a viable alternative [2]. Utilizing drones for ‘last mile’ delivery has thus become technologically feasible, presenting a pressing concern for modern logistics: the efficient and energy-saving utilization of drones in this context. Various scenarios, such as parcel delivery within campuses where human entry is restricted for safety reasons, underscore the inefficiencies of traditional methods like phone calls and text notifications outside campus perimeters, which squander human resources. Consequently, institutions have turned to temporary storage solutions like Cainiao post stations and parcel lockers, though these are limited and susceptible to delays, constraining their broader adoption. The crux of the ‘last mile’ distribution challenge lies in devising energy-efficient routing strategies for drones to optimize logistics operations effectively. Factors influencing drone energy consumption during delivery—such as battery weight, payload capacity, and wind resistance—directly impact operational efficiency [3,4]. While hardware advancements have sought to extend drone flight duration [5], these improvements have plateaued with motor efficiency [6]. Subsequent research has therefore focused on enhancing energy efficiency through improved planning methods, sidestepping the need for major hardware upgrades [7].

In addressing these challenges, researchers have explored various optimization algorithms for vehicle routing problems (VRPs), including genetic algorithms, particle swarm optimization, and ant colony algorithms, etc. these algorithms have achieved good results in routing, but there are still some defects. Ant colony algorithm is a heuristic search algorithm, which has the advantages of positive feedback, parallel computing, and good robustness. However, when using the ant colony algorithm for routing, it is easy to fall into the local optimal situation. Bettinelli et al. [8] designed a branch pricing algorithm to solve the multi-vehicle MDVRP with time window constraint. However, the exact algorithm is not effective in solving complex problems, such as literatures [9,10] adopting different clustering methods to decompose MDVRP into multiple single distribution center VRPs. Ho et al. [11] proposed a hybrid heuristic method combining neighborhood search and genetic algorithm, which uses conservation algorithm Clarke and Wright [12] and nearest neighbor heuristic to generate the initial population, and adopts iterative swap procedure in genetic operation. Nunes Bezerra et al. [13] exploited the scanning algorithm to cluster customer points and generate initial solutions, and designed a method to generate variable neighborhood search to improve the quality of solutions. Wang et al. [14] proposed an improved variable neighborhood search algorithm to solve MDVRP with time window constraints. Xu et al. [15] used the improved optimal cutting algorithm MDVRP split to allocate customers to the distribution center to solve the two-level vehicle routing problem. To solve the emergency logistics problem, Zeng et al. [16] established the cumulative time MDVRP model and proposed a multi-starting point variable neighborhood descent method. Wen and Wu [17] adopted the idea of ”clustering first, routing second”, and proposed a three-stage algorithm to the drone routing problem into subproblems that are easier to solve. Fan et al. [18] constructed a multi-distribution center green vehicle routing problem model and designed an improved ant colony algorithm. To sum up, there are some defects in the current heuristic method: (1) the heuristic method usually adopts the idea of ”grouping first, routing second”, and different groups plan separately, resulting in the loss of overall relevance between groups; (2) In heuristic methods, the quality of grouping usually determines the quality of the overall planning, while the formulation of grouping rules requires a lot of expert neighborhood knowledge, and it is difficult to achieve the optimal effect by artificial grouping rules. Given these limitations, researchers are increasingly turning to deep reinforcement learning (DRL) algorithms [19], propelled by successes like AlphaGo [20], to tackle real-world logistics challenges. Deep Q-network (DQN), an early DRL algorithm, overcame challenges related to sample correlation and convergence speed by leveraging neural networks to approximate action strategies. Despite its effectiveness, DQN’s tendency to overestimate value functions can hinder optimal convergence, particularly in complex environments like MDVRP.

In response, this study proposes an offline routing solution using a multi-agent deep reinforcement learning (EMADRL) approach to minimize drone energy consumption. Specifically tailored to the MDVRP’s complexities and drone energy models, EMADRL integrates deep learning’s attention networks for enhanced feature extraction and decision-making across agents. The study defines the multi-agent reinforcement learning formulation of MDVRP, designs a policy network framework incorporating neighbor structures and masking mechanisms, and introduces novel local search strategies to refine solution quality. This research advances the understanding and application of multi-agent deep reinforcement learning in optimizing ‘last mile’ logistics, promising significant advancements in energy-efficient drone routing and addressing practical challenges in end-to-end delivery logistics.

The contributions of this paper are summarized as follows.

(1) The energy consumption model of drones is comprehensively addressed by developing an energy attenuation model specific to drone operations during package delivery. This model is constructed based on considerations of the drone’s self-weight and the payloads it carries. A linear approximation equation for the power consumption of n-rotor drones is derived and subsequently analyzed to establish a detailed energy consumption framework.

(2) This study introduces EMADRL, a deep reinforcement learning algorithm designed for optimizing drone routing in the context of the multi-depot vehicle routing problem (MDVRP). Initially, the algorithm formulates the problem using a multi-agent MDVRP framework. Next, a policy network model is proposed, specifically tailored to the unique characteristics of the MDVRP, with structured encoder and decoder networks. Subsequently, a strategy network training method is developed to enhance the algorithm’s learning capabilities. Finally, a diverse set of operators is devised to facilitate efficient local search strategies.

(3) The effectiveness of EMADRL is validated through extensive experimentation across multiple scenarios. Empirical results conclusively demonstrate that EMADRL outperforms the representative Deep Deterministic Policy Gradient (DDPG) algorithm in terms of energy efficiency. Specifically, EMADRL achieves average energy savings of 5.96% throughout the drone distribution process across small-scale, medium-scale, and large-scale instances.

The organization of this paper is as follows. Section 2 is a literature review of deep reinforcement learning in drone routing. Section 3 describes the models built, including the drone energy consumption model and MDVRP drone distribution model. Section 4 elaborates the proposed deep reinforcement learning algorithm EMADRL. Section 5 gives detailed experimental results and their analysis. Section 6 summarizes the conclusion of this paper.

## 2. Related Works

In recent years, drone logistics distribution has emerged as a prominent research area, with a significant focus on optimizing drone route planning for enhanced efficiency. However, the issue of energy consumption in drone operations has been addressed in only a limited number of studies. For instance, Thelasingha et al. [21] examined energy-aware UAV-UGV cooperative task site assignments and proposed an iterative planning algorithm tailored to multi-agent systems. Qu et al. [22] investigated environmentally aware and energy-efficient multi-drone coordination strategies in disaster response scenarios.

Research efforts addressing the drone logistics distribution problem with an emphasis on energy consumption typically involve the application of operational research optimization methods or computational intelligence approaches. Recently, scholars have introduced deep reinforcement learning (DRL) methodologies, integrating multiple types of intelligence to optimize these logistics challenges, yielding competitive results. DRL combines the robust representation capabilities of deep learning with reinforcement learning principles, effectively modeling an agent’s action–value or policy functions. This approach is particularly advantageous for tackling the complexities associated with large action and state spaces, which are frequently encountered in intricate environments. The growing interest in DRL has led to its application in solving Combinatorial Optimization Problems (COPs), with the vehicle routing problem (VRP) serving as a prominent example.

Vinyals et al. [23] pioneered the use of a pointer network neural model to address the Traveling Salesman Problem (TSP), marking a significant milestone in the application of deep learning for end-to-end VRP solutions. Unlike traditional supervised learning, which requires optimal TSP solutions as training labels, Bello et al. [24] made a groundbreaking contribution by employing reinforcement learning directly for COPs through DRL. Nazari et al. [25] highlighted the independence of VRP solutions from the order of points, substituting the encoder in point networks with linear embedding layers derived from cyclic neural networks. Building on these foundational advancements, Kool et al. [26] developed a transformer-based graph attention network for the encoder, demonstrating improved efficiency in applying DRL to COPs using a strategy gradient algorithm for training. Alam et al. [27] proposed a multi-agent deep reinforcement learning approach with a joint trajectory control, frequency allocation, and packet routing algorithm. Zou et al. [28] formulated an energy scheduling problem for the prosumer-based urban area and proposed a hierarchical agglomerative clustering-based multi-agent dueling double deep Q-network with a multi-step bootstrapping approach.

Further innovations in this field have explored a variety of methodologies, including the use of graph neural networks. For example, Li et al. [29] employed graph convolutional networks to estimate vertex inclusion probabilities in optimal solutions and utilized tree search techniques to solve multiple COPs. Meanwhile, Nowak et al. [30] applied graph convolutional networks to predict edge selection probabilities, achieving optimal TSP solutions through wave search methods, with training labels sourced from an extension of the Lin–Kernighan–Helsgaun 3 (LKH3) algorithm [31] and trained via supervised learning. Additionally, some researchers have integrated heuristic methods with DRL frameworks to enhance solution quality. Costa et al. [32] proposed a learning based 2-opt local search method, which selects the operation nodes of 2-opt through the network model. Chen et al. [33] introduced NeuRewriter, a local rewriting heuristic based on DRL, which was trained using a Q-Actor–Critic approach. Lu et al. [34] developed the L2I heuristic algorithm, incorporating improved interference operators guided by DRL, achieving results that surpassed established solvers such as LKH3 [31]. Similarly, Wu et al. [35] utilized self-attention mechanisms and the REINFORCE algorithm for historical solution-based offline learning, demonstrating promising advancements in heuristic algorithm development. Falkner and Schmidt-Thieme [36] designed the heuristic algorithm JAMPR by using the deep reinforcement learning method to improve multiple parallel solutions and applied it to CVRP and VRPTW problems. Zhang et al. [37] designed a reinforcement learning hyperheuristic algorithm based on DQN. Collectively, these studies underscore the ongoing evolution of methodologies in drone logistics distribution, particularly in the context of energy efficiency and the integration of advanced computational techniques.

Despite these advancements, existing DRL algorithms exhibit limitations, particularly in high-dimensional or continuous action spaces. V-value-based methods, for instance, maximize actions expected to yield maximum Q-values, often proving inefficient in such settings. Policy Gradient (PG) methods, conversely, focus on learning strategies rather than value functions, adapting model strategies to maximize agent rewards [38]. Actor–critic architectures, such as Deep Deterministic Policy Gradient (DDPG), combine policy-based and value-based learning, leveraging separate network approximations for stable convergence [29]. DDPG, a variant of DPG, enhances complex learning in continuous action spaces, addressing convergence challenges through techniques like soft updates and batch learning [30,31]. This versatility has seen applications in practical scenarios like drone navigation, where continuous control actions are paramount. Omar et al. utilized DDPG to train drones to reach specified targets within a spherical coordinate system [33]. In a similar vein, Ben Zhou et al. employed DDPG for drone navigation and routing tasks, leveraging its capabilities in handling continuous action spaces [34].

## 3. Problem Formulation

### 3.1. Assumptions

The drone routing problem with a focus on energy consumption is delineated as follows: A set of customer nodes with specified demands exists alongside multiple distribution regions. Each distribution region is allocated a drone, each capable of carrying a maximum payload. The geographical coordinates of all customer nodes are known, along with the demand at each customer node. The objective is to minimize the total route distance by optimizing the drone routes. To facilitate the analysis and study of this problem, the following assumptions are posited:

(1) All drones are identical in type and payload capacity.

(2) The demands of customer nodes serviced by a drone do not exceed its payload capacity.

(3) Each customer node is exclusively serviced by a single distribution region.

(4) Each drone commences its journey from a distribution center and returns to the same center after serving multiple customer nodes.

(5) Sub-routes traversed by drones do not feature any internal loop. It is important to note that while the calculated routes for the drone may intersect in actual flight operations, the intersection is limited to the pathways between nodes; customer nodes themselves cannot overlap.

### 3.2. Energy Consumption Model

The energy consumption plays a pivotal role in determining drone endurance, making the accurate estimation of an energy consumption model during customer service critically important. The rate of energy expenditure is contingent upon both the drone’s self-weight and the varying payload it transports. As parcels are delivered to individual customer nodes, the drone’s load progressively decreases, leading to a corresponding reduction in energy usage. During its delivery operation, the drone sequentially loads the assigned parcels and visits the designated customer nodes, unloading each parcel in turn. This step-by-step unloading process results in a gradual reduction in the drone’s payload, which in turn affects its energy consumption dynamics over the course of its delivery route. Therefore, understanding and accurately modeling the relationship between payload reduction and energy consumption is essential for optimizing drone logistics operations.

The energy consumption rate of a drone can be conceptualized as varying linearly with the payload [39,40]. Consequently, the function that estimates this rate is characterized by a segmented form, exhibiting a gradual decline as the payload increases. Building on this foundation, it is possible to calculate the total energy consumed by the drone during an adjacent sub-route, thereby allowing for an assessment of whether the energy expenditure exceeds the battery’s capacity. Additionally, the cost associated with the adjacent sub-route can be derived. For the purposes of this analysis, we neglected the service time and operational costs incurred by the drone at the customer node, as the drone’s dwell time at such nodes is minimal, particularly when a specialized platform is utilized for parcel receipt.

**Figure 1:**
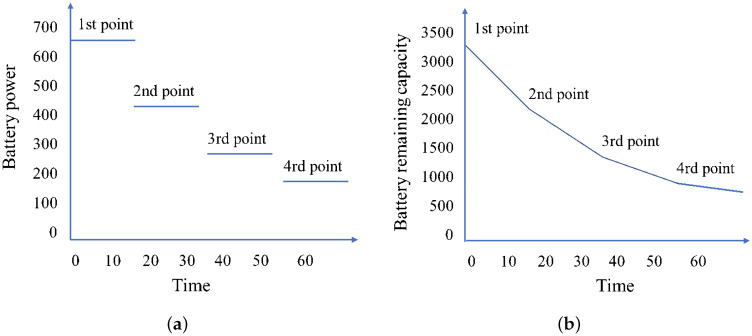
(**a**) Changes in battery power when delivering 4 parcels. (**b**) Change in battery remaining capacity when delivering 4 parcels.

Figure 1a illustrates the fluctuation in battery power of the drone throughout a sub-route involving the delivery of four parcels. This figure delineates four distinct stages of battery consumption, indicating that each parcel delivered corresponds to a specific stage of energy usage. Figure 1b further complements this analysis by depicting the changes in the remaining battery capacity along this route, thus providing a comprehensive overview of the energy dynamics inherent to the drone’s operational framework.

According to the energy consumption model of a drone [40], in the actual operation scenario, its consumed power is approximately linearly proportional to the weight and load of the battery. We calculated the energy consumed by the multi-rotor drone during flight but not during takeoff or landing. We assumed that the drone was a six-axis aircraft, and the single-rotor helicopter’s power P* (in watts) could be calculated as follows:

(1) $$P^{*}=\frac{T^{3/2}}{\sqrt{2\rho \zeta }}$$

where *T* is the thrust (in newtons), ρ is the fluid density of the air (in kg/m^3^), and ζ is the area of rotating blade disc (in m^2^). The thrust T=(W+m)g, given the frame weight *W* in kg, the battery and payload weight *m* in kg, and gravity *g* in N.

According to (1), the equation of the power consumed by the *n*-rotor helicopter can be obtained. Assuming that the total weight of the drone is W+m, and the weight of the battery and load carried by each rotor is m’=m/n, the power consumed by the drone with *n* rotors can be calculated as follows:

(2) $$P={\left(W+m\right)}^{3/2}\sqrt{\frac{g^{3}}{2\rho \zeta n}}$$

The linear approximation equation of the *n*-rotor drone power consumption in Equation (2) can be expressed as p(m)=αm+β, where α represents the energy consumed per kg of battery, *m* is the payload, β is the energy required to keep the six-rotor drone frame in the air. According to the energy consumption model of drones, we can optimize the flight route of a group of drones to minimize the distribution cost of drones. In this model, the linear approximation may be applicable to other multi-rotor helicopters. Parameters *n*, ζ, ρ, and *W* should be similar among different drone types, both of which are under the square root of (2), which means that parameter adjustment has only limited impact on ρ. However, since the energy consumption is exponentially related to the total weight *m* of the drone, a large change in m significantly reduces the accuracy of the approximation.

Therefore, according to Equation (2), the energy consumption model can be derived as:

(3) $$E_{ij}=t_{ij}P=t_{ij}{\left(W+m\right)}^{3/2}\sqrt{\frac{g^{3}}{2\rho \zeta n}}$$

where $t_{ij}$ is the flight time of a drone from customer *i* to customer *j*. On this basis, we can calculate the total energy consumed by the drone in the $t_{ij}$ time period to determine whether it exceeds the battery capacity. Note that we neglected the service time and cost of a drone at the customer node. When there is a special platform to receive packages, the drone stays at the customer node for a short time. Our model and method can also be extended to consider service time and cost by adding relevant parameters.

### 3.3. Mathematical Formulation

Suppose there are *M* distribution centers, and the distribution center set is D=1,2,…,M; there are *N* customer nodes, the customer set is C={1,2,…,N}, and the demand of customer nodes is $\lambda_{i}$, i∈C; The set of all nodes is V=D∪C; the drone set is $K=\{K_{d}\}$, d={1,2,…,D}, $K_{d}$ is the drone set of distribution center *d*, and the total number of drones is L=|K|; the maximum payload of drone is *Q*. The distance between all distribution centers and customer nodes is a Euclidean distance. The MDVRP mathematical model is presented as follows:

(4) $$\min\;\sum_{d\in D}\sum_{k\in K_{d}}\sum_{i\in V}\sum_{j\in V}Dis\left(i,j\right)\times x_{ijk}$$

(5) $$s.t.\;\sum_{i\in V}x_{ijk}=\sum_{i\in V}x_{jik},\;\forall j\in C,\;k\in K$$

(6) $$\sum_{k\in K}\sum_{j\in V}x_{ijk}=1,\;\forall i\in C$$

(7) $$\sum_{i\in C}\lambda_{i}\sum_{j\in V}x_{ijk}\leq Q,\;\forall k\in K$$

(8) $$\sum_{d\in D}y_{kd}=1,\;\forall k\in K$$

(9) $$\sum_{d\in D}\sum_{k\in K}y_{kd}\leq L\;$$

(10) $$x_{ijk}\in \left\{0,1\right\},\;\forall i,j\in V,\;k\in K\;$$

(11) $$y_{kd}\in \left\{0,1\right\},\;\forall k\in K,d\in D\;$$

Constraint (4) is the objective function to minimize the total length of the transportation route. Constraint (5) indicates that the number of entry and exit sides of all customer nodes is equal. Constraint (6) denotes that all customer nodes only have one drone service; meanwhile, Constraints (5) and (6) ensure that the number of entry and exit sides of all customer nodes is 1. Constraint (7) imposes that the customer demands serviced by a drone shall not exceed its maximum payload. Constraint (8) restricts that each drone belongs to the distribution center uniquely. Constraint (9) restricts that the total number of all drones shall not exceed the maximum number of drones. Constraint (10) is the decision variable. Constraint (11) is the drone ownership decision variable.

## 4. The Proposed EMADRL Algorithm

As each route state selection of distribution drone is not continuous but discrete, we should build a mathematical model for the random state transfer, discrete traveling state, and action selection mechanism of drones according to reinforcement learning. First, the multi-agent reinforcement learning form of MDVRP is defined; second, a strategy network based on the encoder–decoder structure is designed, and the network model is trained by the strategy gradient reinforcement learning method; third, different action selection strategies and search strategies are adopted to obtain higher-quality solutions.

### 4.1. Multi-Agent Reinforcement Learning Form of MDVRP

In constructing the routing problem model of multiple drones, the state space, action space, state transition, and return function of the MDVRP are defined, and the multi-agent reinforcement learning form of the MDVRP is formed.

State space: State space $S=S_{g},S_{a}$ is divided into global state $S_{g}$ and agent state $S_{a}=S_{a,1},S_{a,2},\ldots ,S_{a,D}$. The global state $S_{g}$ is a static state. The agent state $S_{a}$ is composed of the states of all agents. The state of a single agent *d* is $S_{a,d}=l_{d};r_{d}$. $l_{d}$ is the node feature selected by agent *d* in the previous step. $r_{d}$ is the current drone’s remaining capacity of agent *d*. $S_{a}$ and *d* are dynamic states and change with time.

Action space: The multiple-action space is the joint action space of all agents $A_{t}=A_{d}^{t}$, where d=1,2,…,D. The agent action $A_{d}^{t}$ is the node selected by agent *d* at the current time step *t*, including the customer node that has not been visited and the distribution center point corresponding to the agent.

State transition: At the conclusion of the current time step *t*, an action, denoted as $A_{d}^{t}$, is selected by agent *d*. The agent’s state then transitions according to the formula $S_{a,d}^{t}=S_{a,d}^{t-1}*A_{d}^{t}$, where the symbol ‘*’ represents the addition of the customer node selected by the action to the agent’s current state. This process continues incrementally until the full joint action, $a_{t}$, is formed. Consequently, the complete system state transitions from the previous state $S^{t-1}$ to the updated state $S^{t}$, reflecting the accumulation of actions over the time step. This transition mechanism is integral to capturing the dynamic progression of the agent’s decision-making process.

Reward function: For the MDVRP, the objective function is to minimize the total distribution distance. The smaller the total distance, the higher the accumulated return of the agent. Therefore, the negative number of the total distance is taken as the accumulated reward *R*, which can be expressed as follows:

(12) $$R=-\sum_{d=1}^{D}\sum_{t=1}^{T-1}Dis(A_{d}^{t},\;A_{d}^{t+1})$$

where $A_{d}^{t}$ represents the action chosen by agent *d* at the time step *t*, where the action is chosen from the customer node set. Dis represents the distance between the nodes corresponding to the two states of $S^{t-1}$ and $S^{t}$ at time step t−1 and time step *t*. The sum of the distances between these nodes constitutes the total distance of agent *d*, where d∈{1,2,…,D}, and the total distance of all agents constitutes the reward function *R*.

The $\pi_{\theta }$ operates by selecting an action $A_{t}$ at each time step *t*, continuing until the terminal state is reached. The action at each step is determined by the probability vector $p_{\theta }$, which is produced by the policy network. This strategy produces a complete sequence of nodes, represented as $\pi =\pi_{1},\pi_{2},\ldots ,\pi_{T}$, where *T* denotes the length of the customer node sequence. Thus, the probability of generating a complete strategy π for a given instance *s* is calculated as a product of the individual probabilities associated with each action in the sequence. This structured approach enables the modeling of decision-making processes under uncertainty while adhering to the policy’s probabilistic framework.

(13) $$P\left(\pi |s\right)=\prod_{t=1}^{T}p_{\theta }\left(\pi_{t}|s_{t-1},\;\pi_{t-1}\right)$$

### 4.2. The Proposed EMADRL Algorithm

#### 4.2.1. EMADRL Policy Network Model

The strategy network proposed in EMADRL consists of an encoder and a decoder. The encoder processes the input data to generate a hidden state that encapsulates the feature information of the entire graph. Meanwhile, the decoder outputs the probability distribution over actions at each time step, enabling the selection of specific actions based on these probabilities. This process iteratively updates the state until reaching a terminal state.

**Figure 2:**
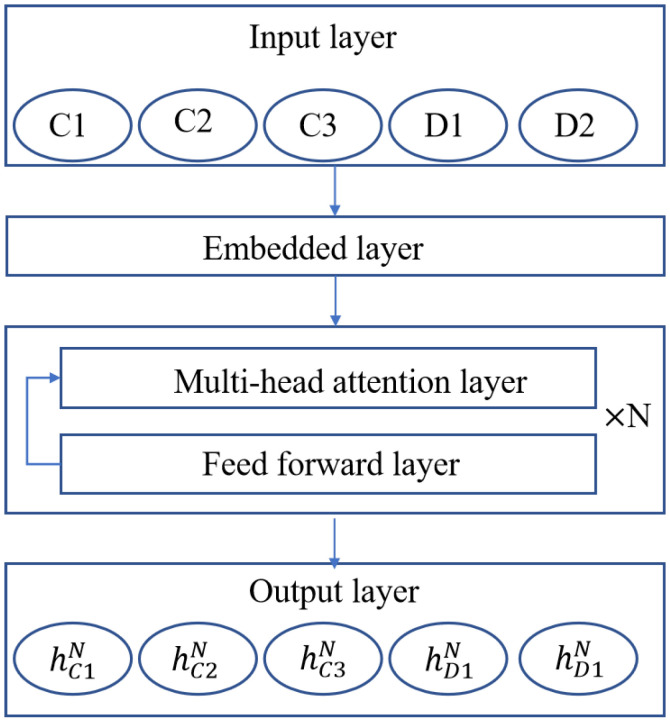
The encoder network structure.

The encoder architecture in EMADRL, as depicted in Figure 2, is comprised of several integral components: an input layer, an embedding layer, *N* attention modules, and an output layer. The input layer accommodates the customer nodes as well as the distribution center node, while the output layer generates a high-level feature representation that encapsulates both individual node attributes and overarching graph feature information. Within the *N* attention modules, each module maintains an identical structural configuration but operates with independent network parameters. Each attention module consists of a multi-head attention layer (MHA) and a feed-forward layer (FF), facilitating the encoder’s ability to effectively capture individual node characteristics alongside the broader relational dynamics within the graph. This design enhances the model’s capacity for a nuanced understanding of the system’s underlying dynamics and interactions.

Specifically, the embedding layer takes the characteristics of distribution center points and customer points as the input $X=x_{i};\forall i\in V,x_{i}=\;[cx_{i};cy_{i};\lambda_{i}],$ where ‘;’ denotes the splicing between different features, cx and cy are the abscissa and ordinate of the node in the plane coordinate system, and $\lambda_{i}$ is the distribution demand of the node. The embedded layer maps each input $x_{i}$ to the node embedding feature $h_{i}^{0}$, which has

(14) $$h_{i}^{0}=W^{X}\times x_{i}+b^{x}$$

**Figure 3:**
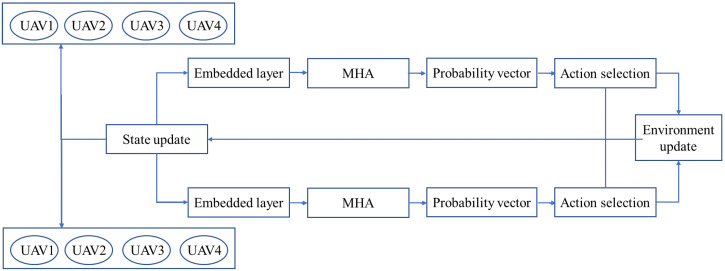
The decoder network structure.

The decoder in EMADRL is the multi-agent framework that uses the state information $S_{t-1}$ = $S_{g}$; $S_{a_{t}-1}$ of the previous time step to obtain the context vector, outputs the selection probability vector of the current time step action, and selects the next action through the selection strategy. The decoder network structure is shown in Figure 3.

The multi-head attention layer of the decoder uses the masking mechanism to mask the inaccessible nodes and calculates the scaling point multiplication vector between query and key. The normalized attention score is obtained by using the softmax function. The updated context vector is qdc. The masking mechanism is described as follows: for each node j∈V, if it does not meet any of the following conditions, it is masked: (1) *j* is the neighbor node of the distribution center corresponding to the current agent; (2) node *j* is not accessed; (3) the demand of *j* is less than the remaining capacity of the corresponding vehicle of the current agent.

The decoder selects the probability vector through a single attention layer output node. The attention layer calculates the compatibility degree between the query and the key. The query is calculated from the context vector updated by multiple attention layers, and the key is calculated from the node characteristics. We normalize with the softmax function to obtain the selection probability of each node. According to the probability vector $p_{d,j}$ output by each agent, the next action $A_{d}^{t}$ of the agent is selected by the action selection strategy, and the joint action $A^{t}=A_{1}^{t},A_{2}^{t},\ldots ,A_{D}^{t}$ is obtained when all customers have visited, forming a complete policy solution $\pi =\pi_{1},\pi_{2},\ldots ,\pi_{T}$.

#### 4.2.2. EMADRL Strategy Network Training Method

Building on the principles of the REINFORCE algorithm with a baseline, the policy gradient for a single agent is computed, forming the foundation for training an individual agent’s strategy. The policy gradient is then calculated using the REINFORCE algorithm with a baseline, and the parameters of the policy network are updated through gradient descent to optimize performance, which can be expressed as follows:

(15) $$\nabla L\left(\theta |s\right)=-E_{p\theta (\pi |s)}\left[\left(R\left(\pi \right)-R\left(\pi^{bl}\right)\right)\times \nabla_{\theta }\log p_{\theta }\left(\pi |s\right)\right]$$

(16) $$\theta =Adam(\theta ,\;\nabla_{\theta }L\left(\theta |s\right))$$

Incorporating the reference network $\theta^{bl}$ plays a crucial role in reducing the variance of the gradient during network training, thereby stabilizing the learning process. The reference network, serving as a performance benchmark, is updated through a rollback mechanism, while the policy network is updated at the conclusion of each training round. This rollback ensures that the reference network reflects the improved policy, contributing to more effective training.

#### 4.2.3. EMADRL Local Search Strategy

The trained EMADRL demonstrated rapid solving capability for MDVRP through a greedy action selection strategy. However, there was scope for enhancement, particularly in challenging instances characterized by issues such as sub-route crossings and the tendency of the greedy strategy towards overconfidence, occasionally leading to the omission of actions with high selection probabilities. To elevate the solution quality, two distinct local search strategies were integrated into EMADRL.

(1) Two-opt search: For the route-crossing problem in the sub-loop, the 2-opt local search takes each sub-loop of the solution output by the model as the initial solution and performs the 2-opt operation on all sub-loops to optimize, so as to improve the overall quality of the solution. The specific steps are as follows.

Step 1: randomly select two nodes of the current sub-loop *r*, and flip the route between the node pairs to form a new sub-loop $r^{\prime }$. Step 2: if the sub-loop $r^{\prime }$ is better than *r*, update the current sub-loop $r=r^{\prime }$, reset the iteration number iter to 0 and return to step 1; otherwise, the iter = iter + 1 and return to step 1. Step 3: if the number of iterations reaches the maximum number of iterations, iter = MaxIter, and *r* is not improved, the 2-opt local search is ended, and *r* is returned as the optimal sub-loop.

(2) Sampling search: Specifically, if *s* represents the number of solution samples, the optimal solution is determined as $\pi^{*}$ = *argmin*$\left(L\left(\pi_{1}\right),L\left(\pi_{2}\right),\ldots ,L\left(\pi_{s}\right)\right)$, where $L(\pi_{i})$ denotes the cost of each solution $\pi_{i}$. This approach mitigates the risk of overconfidence in the policy network by leveraging the diversity of sampled solutions. Given the model’s ability to rapidly generate solutions, the repeated sampling process incurs minimal time cost, making it a computationally efficient strategy for identifying optimal results.

After multiple training iterations, the strategy network exhibited improved decision-making capabilities. The action selection was governed by the probability vector output from the network, employing two distinct strategies: (1) The greedy action selection strategy, which placed full trust in the policy network. At each step, it selected the action with the highest probability as indicated by the decoder’s output probabilities. (2) The sampling action selection strategy, which treated the decoder’s output probabilities as a sampling distribution, and selected actions through random sampling.

## 5. Experiments and Analysis

### 5.1. Experiment Settings

To evaluate the effectiveness of the proposed EMADRL algorithm, we conducted training across three distinct scales: small scale (30-2 configuration with 30 customer nodes and 2 distribution center nodes), medium scale (50-3 configuration with 50 customer nodes and 3 distribution center nodes), and large scale (100-5 configuration with 100 customer nodes and 5 distribution center nodes). Each scale comprised five instances generated using the CVRP instance method detailed in [26], where customer node demands were uniformly distributed over the interval [0, 10]. The experiments were conducted on a single GPU, operating under Windows 10 with an Intel Core i7 CPU running at 3.80 GHz.

During the model training phase, we set the number of epochs to 100, with 500 training batches per epoch and 512 instances per batch. In the sampling search process, each instance was sampled 128 times. We employed the Adam optimizer [41] with a learning rate of 1 × $10^{-4}$ to optimize the network parameters governing the strategy. For instance testing, we evaluated 10,000 test groups under their respective distributions. Model performance was assessed based on the average path length and average solution time across all test instances, with shorter route lengths indicating superior strategy performance and reduced energy consumption. Detailed hyperparameter configurations for both the EMADRL and DDPG algorithms can be found in Table 1.

### 5.2. Comparison and Analysis of Algorithms

**Figure 4:**
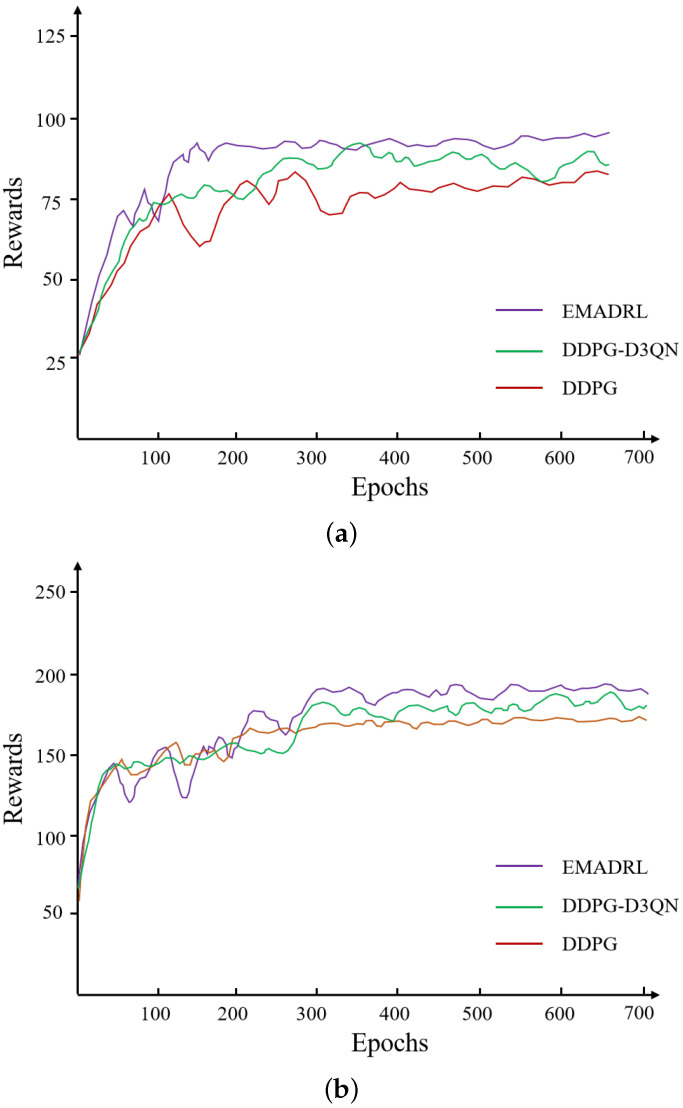

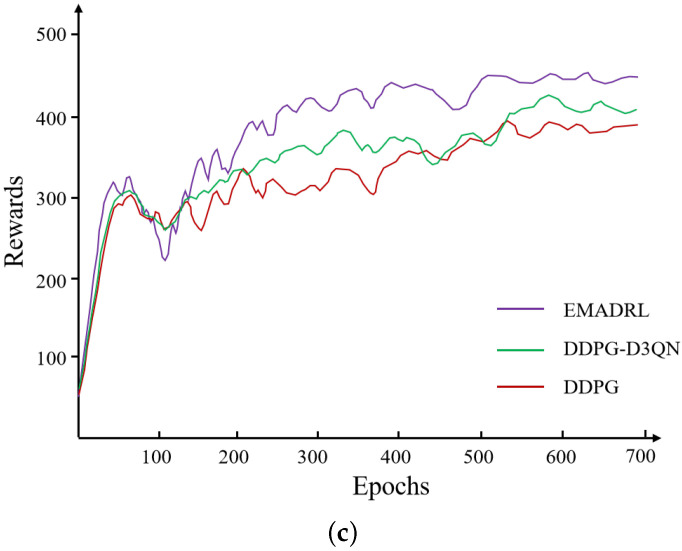
The initial solution schematic diagram by combining the nearest neighbor and cost-savings strategies. (**a**) Reward values curve on CVRP 30. (**b**) Route length curve on CVRP 50. (**c**) Reward value curve on CVRP 100.

To visually illustrate the learning progression and convergence characteristics of the proposed EMADRL algorithm in comparison to both the DDPG algorithm and its enhanced variant, the DDPG-D3QN algorithm, we present the reward curves and optimization objective function values across small-, medium-, and large-scale instances in Figure 4. In this analysis, the objective function values represent the total distance cost incurred by the drone during its operations. A lower distance cost is associated with reduced energy consumption, which indicates improved algorithmic efficacy and effectiveness in logistics distribution tasks.

To enhance the clarity and interpretability of the experimental outcomes, we employed a smoothing technique that averaged the reward and optimization objective function values over every 20 steps. This method reduced noise and fluctuations inherent in the raw data, thereby facilitating a more coherent analysis of performance trends across the different algorithms. The representations in Figure 4 thus provide valuable insights into the comparative strengths and weaknesses of the EMADRL algorithm, highlighting its potential advantages in optimizing drone logistics operations effectively.

Figure 4 presents the typical deep reinforcement learning curves for the EMADRL, DDPG-D3QN, and DDPG algorithms. The reward curve of the agent exhibited fluctuations and an upward trend, indicating the adaptive adjustment of action selection strategies based on accumulated learning experiences. Initially, the EMADRL curve demonstrated significant fluctuations, which could be attributed to lower Q-value estimation accuracy during the early stages of training. However, as training progresses, the acquisition of more effective strategies led to stabilized fluctuations, underscoring the convergence and precision achieved by both deep reinforcement learning methods.

Furthermore, Figure 4 illustrates that EMADRL achieved solution qualities comparable to those of the competing algorithms in the 30-2 and 50-3 instances, while significantly outperforming them in terms of solution time. In the 100-5 instances, EMADRL exhibited superior performance relative to both the DDPG-D3QN and DDPG algorithms in terms of both solution quality and computational efficiency. Notably, EMADRL’s integration of two-opt local search and sampling strategies proved advantageous across all three scales, enhancing solution quality and efficiency. A comparative analysis of these strategies revealed that the sampling approach, although it did not always select the action with the highest probability from the policy network, effectively improved solution quality by probabilistically favoring actions with higher likelihoods. This observation emphasizes the efficacy of incorporating sampling strategies alongside local search techniques to enhance solution outcomes in diverse operational contexts.

When comparing EMADRL with the DDPG and DDPG-D3QN algorithms in the CVRP-30 instances, both algorithms demonstrated the ability to explore better solutions and achieve rapid convergence on a smaller scale. In the CVRP-50 and CVRP-100 instances, while the DDPG algorithm exhibited a rapid convergence trend, it suffered from exploration limitations and overestimation issues, leading to an overreliance on previously explored action strategies. This dependence resulted in only marginal improvements in the final convergence value. A statistical analysis revealed that the average distance costs for EMADRL across the three scales—CVRP-30, CVRP-50, and CVRP-100—were 6.31, 11.02, and 15.95, respectively, which were 1.92%, 1.27%, and 2.08% higher than those of the DDPG algorithm, thus yielding competitive experimental results.

Although the convergence time for the proposed EMADRL algorithm was longer than that of the DDPG and DDPG-D3QN algorithms, it is important to note that in actual application environments that leverage the higher computational power of GPU servers or supercomputing platforms, EMADRL could rapidly produce feasible solutions of higher quality. In conclusion, the proposed EMADRL consistently achieved higher rewards and lower costs across small-scale, medium-scale, and large-scale instances, demonstrating its effectiveness in optimizing logistics distribution challenges.

### 5.3. Sensitivity Analysis of EMADRL

To further investigate the impact of algorithm hyperparameters in the EMADRL, we conducted a sensitivity analysis on three critical parameters: the discount rate factor, the learning rate factor, and the mini-batch size.

#### 5.3.1. Sensitivity Analysis of Discount Rate

The appropriate setting of the discount rate is crucial for determining the expected reward value associated with each state in reinforcement learning. In this study, while maintaining other parameters constant, we systematically varied the discount factor γ among values of 0.7, 0.9, and 0.99. The experimental results indicated that the adjustment of the discount rate γ had a significant impact on both the variation and convergence of the optimization objective value.

Specifically, when γ was set to 0.99, the optimization objective failed to approach its optimal level. This phenomenon could be attributed to an excessive emphasis on past experiences, which hindered the model’s ability to adapt to new information effectively. In contrast, a lower discount rate of 0.7 limited the model’s capacity to learn optimal action selections from historical data, resulting in a protracted convergence process within the specified iteration steps.

Interestingly, the optimal convergence speed was observed at γ = 0.9, which stroke a balanced equilibrium between valuing immediate rewards and acknowledging the importance of future rewards. This setting allowed the model to effectively leverage historical data while remaining responsive to new experiences. Consequently, we selected γ = 0.9 for the EMADRL algorithm due to its favorable convergence characteristics, which facilitated efficient learning and optimal decision-making in complex environments.

#### 5.3.2. Sensitivity Analysis of Learning Rate

The appropriate setting of the learning rate is critical for determining the convergence behavior of an algorithm. In this study, we maintained all other parameters constant while systematically varying the learning rate α among values of 0.005, 0.05, and 0.001. The experimental results revealed that adjustments to the learning rate α significantly affected both the convergence speed and stability of the optimization objective.

Specifically, setting α to either 0.05 or 0.005 facilitated rapid initial convergence; however, this often led to instability and failure to accurately learn the optimal strategy, resulting in subsequent oscillations in the optimization trajectory. In contrast, a lower learning rate of 0.001 allowed for a more gradual and steady improvement of the optimization target, demonstrating enhanced convergence properties. This stability was crucial for ensuring that the algorithm did not diverge or oscillate excessively, which could impede effective learning.

Consequently, we selected α = 0.001 for the learning rate in the EMADRL algorithm. This choice stroke a favorable balance between convergence speed and stability, thereby ensuring effective learning and reliable convergence toward optimal strategies in complex environments. By adopting this learning rate, we enhanced the algorithm’s robustness, allowing it to effectively navigate the trade-offs inherent in reinforcement learning tasks.

#### 5.3.3. Sensitivity Analysis of Mini-Batch Size

The proper setting of the mini-batch size determines the training efficiency and performance. Keeping other parameters unchanged, the mini-batch size β values were set to 32, 64, and 128, respectively.

By employing multiple instances for drone logistics routing problems, we could ascertain the optimal mini-batch size of the EMADRL algorithm that balanced computational efficiency and generalization capabilities. The analysis involved varying the mini-batch size across a predefined range while monitoring key performance metrics, including training loss, validation accuracy, and convergence time. The results of this sensitivity analysis indicated a distinct trade-off between convergence speed and model stability based on the selected mini-batch size in the EMADRL algorithm. Smaller mini-batches (β = 32) tended to lead to more rapid updates of model parameters, which could accelerate initial convergence; however, they often introduced a higher degree of stochasticity into the training process. This noise could destabilize learning, leading to oscillations in loss values. Conversely, larger mini-batches (β = 128) generally produced more stable and accurate gradient estimates, resulting in smoother convergence trajectories. Nevertheless, this came at the cost of slower overall convergence rates, as fewer updates were made per epoch. Thus, determining the best mini-batch size of β = 64 became a balancing act between the benefits of speed and stability.

Furthermore, the sensitivity analysis revealed that mini-batch size significantly influenced the EMADRL algorithm’s ability to generalize to unseen data. Smaller mini-batch sizes introduced a regularizing effect due to the inherent noise, which could help mitigate overfitting by encouraging exploration of the loss landscape. In contrast, larger mini-batches could lead to overfitting, as the drone routing model was more likely to learn noise and specific patterns from the training data. This aspect underscores the importance of empirical validation. Consequently, practitioners are encouraged to conduct sensitivity analyses as a fundamental step in their model training pipeline, ensuring that a mini-batch size of 64 is conducive to both efficient training and robust generalization.

### 5.4. Energy-Saving Comparison and Analysis

To further explain the energy-saving effect of the proposed EMADRL based on deep reinforcement learning, Table 2 shows the energy consumption and path length results of multiple drones on small-scale, medium-scale, and large-scale instances. All of these results are compared with the experimental results of DDPG.

From Table 2, compared with DDPG, EMADRL saved 5.22%, 6.16%, 5.48%, 4.38%, and 11.16% in energy consumption on the five small-scale instances, respectively. Among them, the fifth instance showed the largest energy saving, i.e., a saving of 11.16% in energy consumption. On the medium scale, EMADRL saved 3.12%, 6.07%, 5.26%, 12.45%, and 3.69% in energy consumption, and the fourth instance showed an energy saving of 12.45%, which was the largest energy saving on the medium-scale instances. On the large-scale instances, the path optimized by EMADRL saved 3.06%, 3.32%, 5.58%, 7.54%, and 6.84% in energy consumption, respectively, where the fourth instance showed the maximum energy consumption saving of 7.54%. In all instances affected by the above three scales, the maximum energy saving reached was 12.45%.

Additionally, the proposed EMADRL achieved average energy savings of 6.48%, 6.12%, and 5.27% on small-scale, medium-scale, and large-scale instances, respectively. Across all instances of these scales, the overall average energy savings amounted to 5.96%. The experimental findings indicated that while DDPG failed to guarantee optimal multi-drone delivery solutions, EMADRL incorporated an encoder tailored for MDVRP characteristics and integrated heuristic operators for local search. This approach enabled drones to efficiently navigate routes that minimized energy consumption based on learned models.

The observed differences could be attributed to the potential interference among multiple drone distribution routes generated by DDPG, impacting energy efficiency. Moreover, centralized routing solutions often require iterative calculations to achieve optimal routes. In contrast, EMADRL’s integrated approach during training accounts for such interference without necessitating additional adjustment models.

In summary, based on these experimental outcomes, EMADRL achieved an average energy saving of 5.96% across all scales compared to DDPG, with maximum savings reaching 12.45%. The optimized routes derived from EMADRL more closely approximated theoretical optimal values, demonstrating superior performance over DDPG in comparable-scale instances and enhancing the energy efficiency of drone delivery operations.

## 6. Conclusions

In this study, we developed an energy consumption model for drone routing that incorporated the relationship between drone weight, payload, and energy expenditure during parcel delivery. Building upon this model, we proposed a multi-agent framework based on deep reinforcement learning for optimizing drone routing. By leveraging interactions between drones and customers, our approach derived rewards to guide strategic action combinations, addressing end-to-end routing challenges while adhering to payload constraints. Following extensive training, our algorithm demonstrated significant enhancements in efficiency for solving drone routing problems.

The proposed algorithm achieved notable energy savings, with up to 12.45% improvement observed in individual instances and an average energy saving of 5.96% across all three scale scenarios. This model and algorithm introduce novel concepts and methodologies applicable to modern logistics applications, such as smart campus logistics distribution. Future research will explore multi-objective optimization algorithms to further refine energy consumption considerations across diverse operational factors.

## Figures and Tables

**Table 1:** The hyperparameters of the EMADRL and DDPG algorithm.

	DDPG	EMADRL		DDPG	EMADRL
**Actor**	**Value**		**Hyperparameter**	**Value**	
Layer size	[state, 500, 500, action]		Actor learning rate	0.0005	
Activation function	ReLU		Critic learning rate	0.005	
Optimizer	Adam		Target update factor	0.005	
Critic	Value		Mini-batch size	512	
Layer size	[state, 500, 500, q-value]		Memory buffer size	1,000,000	
Activation function	ReLU		Discount factor	0.98	
Optimizer	Adam		Policy update delay		3
Critic 2	N	Y	Exploration noise	-	0.2

**Table 2:** The comparison of energy consumption between the proposed EMADRL and DDPG algorithms.

		Energy Consumption (kJ)				Travel Dis.	
Scale	Instance	DDPG	EMADRL	Energy Saving (%)	Avenge (%)	DDPG	EMADRL
Small	1	127.63	121.3	5.22	6.48	36.51	32.94
	2	132.82	125.11	6.16		33.39	30.41
	3	139.58	132.33	5.48		38.62	29.84
	4	126.94	121.61	4.38		32.27	31.55
	5	132.81	119.48	11.16		37.28	30.79
Medium	1	247.1	239.62	3.12	6.12	53.17	50.63
	2	229.51	216.37	6.07		49.20	45.85
	3	235.27	223.52	5.26		48.96	47.56
	4	226.59	201.5	12.45		51.38	48.2
	5	218.29	210.53	3.69		52.32	49.48
Large	1	349.56	339.17	3.06	5.27	83.68	80.42
	2	329.62	319.04	3.32		84.92	75.93
	3	333.48	315.85	5.58		79.14	76.9
	4	325.33	302.51	7.54		81.81	78.69
	5	347.89	325.63	6.84		79.97	74.21

## Data Availability

The original contributions presented in the study are included in the article, further inquiries can be directed to the corresponding author.
